# (2*R*,3*R*)-3-(2-Chloro­phen­yl)-*N*-phenyl­oxirane-2-carboxamide

**DOI:** 10.1107/S1600536809048442

**Published:** 2009-11-21

**Authors:** Lian-Mei Chen, Tai-Ran Kang

**Affiliations:** a College of Chemistry and Chemical Engineering, China West Normal University, Nanchong 637002, People’s Republic of China

## Abstract

In the title compound, C_15_H_12_ClNO_2_, the two benzene rings adopt a *syn* configuration with respect to the ep­oxy ring; the dihedral angles between the ep­oxy ring and the two benzene rings are 59.71 (16) and 67.58 (15)°. There is a weak intra­molecular N—H⋯O bond, which may help to establish the conformation. In the crystal, the mol­ecules are linked into a chain parallel to the *b* axis through inter­molecular N—H⋯O hydrogen bonds.

## Related literature

For the use of epoxide-containing compounds as building blocks in the synthesis of biologically active compounds, see: Flisak *et al.* (1993 ▶); Porter & Skidmore (2000 ▶); Shing *et al.* (2006 ▶); Watanabe *et al.* (1998 ▶); Zhu & Espenson (1995 ▶). For the isostructuralbromo compound, 3-(2-bromo­phen­yl)-*N*-phenyl­oxirane-2-carboxamide, see: He *et al.* (2009 ▶). For related structures, see: He (2009 ▶); He & Chen (2009 ▶).
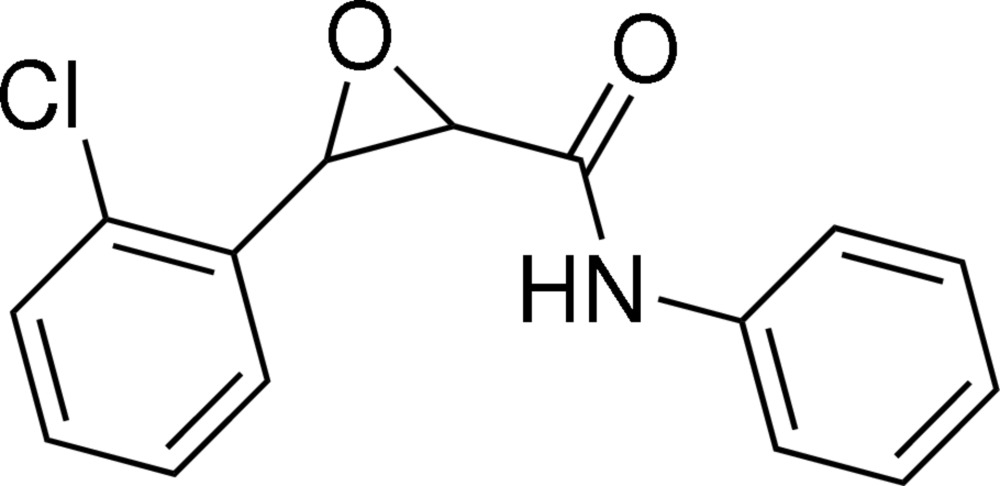


## Experimental

### 

#### Crystal data

C_15_H_12_ClNO_2_
*M* *_r_* = 273.71
Orthorhombic, 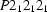

*a* = 6.6610 (1) Å
*b* = 10.0343 (2) Å
*c* = 20.2433 (3) Å
*V* = 1353.03 (4) Å^3^
*Z* = 4
Cu *K*α radiation
μ = 2.48 mm^−1^
*T* = 295 K
0.36 × 0.32 × 0.30 mm

#### Data collection

Oxford Diffraction Gemini S Ultra diffractometer
Absorption correction: multi-scan (*CrysAlis Pro*; Oxford Diffraction, 2009 ▶) *T*_min_ = 0.469, *T*_max_ = 0.524
9086 measured reflections
2373 independent reflections
2100 reflections with *I* > 2σ(*I*)
*R*_int_ = 0.026

#### Refinement

*R*[*F* ^2^ > 2σ(*F* ^2^)] = 0.031
*wR*(*F* ^2^) = 0.109
*S* = 1.20
2373 reflections
172 parameters
H-atom parameters constrained
Δρ_max_ = 0.15 e Å^−3^
Δρ_min_ = −0.26 e Å^−3^
Absolute structure: Flack (1983 ▶), 864 Friedel pairs
Flack parameter: 0.01 (2)

### 

Data collection: *CrysAlis Pro* (Oxford Diffraction, 2009 ▶); cell refinement: *CrysAlis Pro*; data reduction: *CrysAlis Pro*; program(s) used to solve structure: *SHELXS97* (Sheldrick, 2008 ▶); program(s) used to refine structure: *SHELXL97* (Sheldrick, 2008 ▶); molecular graphics: *ORTEPIII* (Burnett & Johnson, 1996 ▶), *ORTEP-3 for Windows* (Farrugia, 1997 ▶) and *PLATON* (Spek, 2009 ▶); software used to prepare material for publication: *SHELXL97*.

## Supplementary Material

Crystal structure: contains datablocks global, I. DOI: 10.1107/S1600536809048442/dn2512sup1.cif

Structure factors: contains datablocks I. DOI: 10.1107/S1600536809048442/dn2512Isup2.hkl

Additional supplementary materials:  crystallographic information; 3D view; checkCIF report

## Figures and Tables

**Table 1:** Hydrogen-bond geometry (Å, °)

*D*—H⋯*A*	*D*—H	H⋯*A*	*D*⋯*A*	*D*—H⋯*A*
N1—H1⋯O1	0.86	2.38	2.801 (3)	111
N1—H1⋯O2^i^	0.86	2.16	2.973 (2)	158

Symmetry code: (i) 
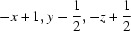
.

**Fig. 1.**
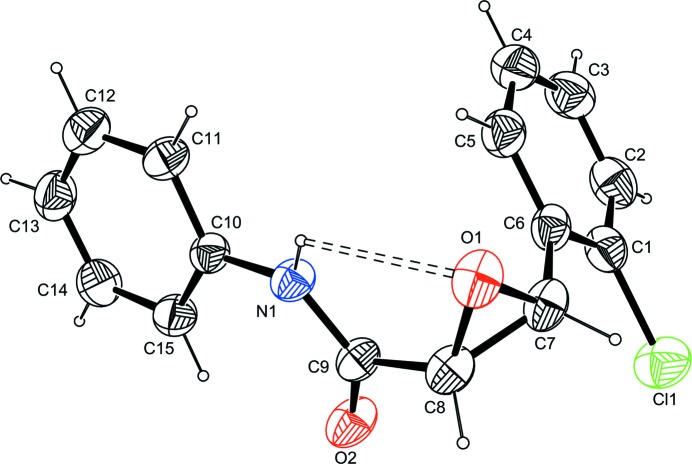
The molecular structure of (I) with the atom labeling scheme. Ellipsoids are drawn at the 30% probability level. H atoms are represented as small spheres of arbitrary radii. Intramolecular hydrogen bond is shown as dashed line.

**Fig. 2.**
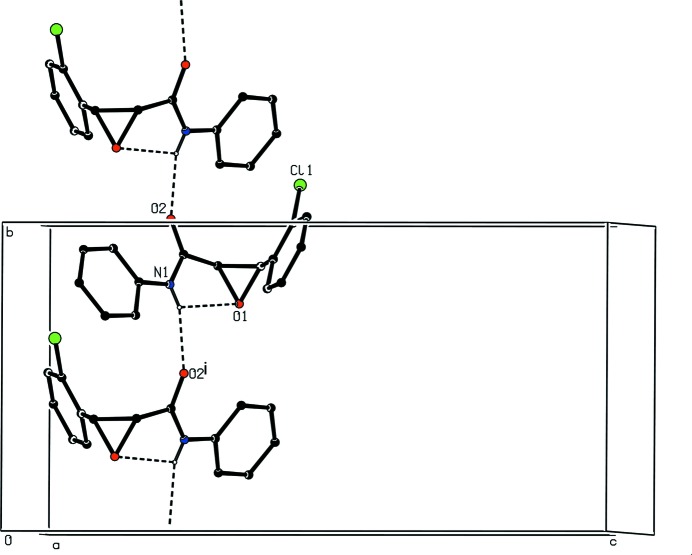
Partial packing view showing the formation of infinite chain parallel to the b axis through N-H···O hydrogen bonds. H atoms not involved in hydrogen bondings have been omitted for clarity. H bonds are shown as dashed lines. [Symmetry code: (i) -x+1, y-1/2, -z+1/2 ]

## supplementary crystallographic information

### Comment

Optically active epoxides are highly useful intermediates as building blocks for the synthesis of biologically active compounds. They can be further transformed to key intermediates of several pharmaceutical products (Flisak *et al.* 1993; Porter & Skidmore, 2000; Watanabe *et al.* 1998; Shing *et al.*, 2006). Various effective systems have been developed over the years for the preparation of chiral epoxides. The Darzens reaction, has proven to be one of the most powerful approaches (Zhu & Espenson,1995). We report herein the crystal structure of the title compound.

The title compound is isostructural of the related bromo compound, 3-(2-Bromophenyl)-N-phenyloxirane-2-carboxamide (He *et al.*, 2009). The two phenyl rings adopt a *syn* configuration with respect to the epoxy ring (Fig. 1). The dihedral angle between the C1—C6 and C10—C15 is 76.27 (7)° and the O1/C7/C8 epoxide ring makes dihedral angles of 59.71 (16)° and 67.58 (15)° with C6 and C15 phenyl ring, respectively, These values are very similar to those observed in related structures (He, 2009; He & Chen, 2009; He *et al.*, 2009).

There is a weak intramolecular N-H···O bond which might induce the observed conformation. The molecules are linked into a chain parallel to the b axis through intermolecular N-H···O hydrogen bonds (Table 1, Fig. 2).

### Experimental

2-chloro-*N*-phenylacetamide (0.17 g, 1.0 mmol) and potassium hydroxide (0.112 g, 2.0 mmol) were dissolved in acetonitrile (4 ml). To the solution was added 2-chlorophenylaldehyde (0.14 g, 1.0 mmol) at 298 K, the solution was stirred for 2 h and removal of solvent under reduced pressure, the residue was purified through column chromatography. Colourless single crystals of (I) were obtained by recrystallization from an ethanol solution.

### Refinement

All H atoms attached to C atoms and N atom were fixed geometrically and treated as riding with C—H = 0.93 Å and N—H = 0.86 Å with U_iso_(H) = 1.2U_eq_(C or N)

### Crystal data

**Table:**

C_15_H_12_ClNO_2_	*F*(000) = 568
*M**_r_* = 273.71	*D*_x_ = 1.344 Mg m^−^^3^
Orthorhombic, *P*2_1_2_1_2_1_	Cu *K*α radiation, λ = 1.54184 Å
Hall symbol: P 2ac 2ab	Cell parameters from 9390 reflections
*a* = 6.6610 (1) Å	θ = 4.4–72.1°
*b* = 10.0343 (2) Å	µ = 2.48 mm^−^^1^
*c* = 20.2433 (3) Å	*T* = 295 K
*V* = 1353.03 (4) Å^3^	Block, colourless
*Z* = 4	0.36 × 0.32 × 0.30 mm

### Data collection

**Table:**

Oxford Diffraction Gemini S Ultra diffractometer	2373 independent reflections
Radiation source: Enhance Ultra (Cu) X-ray Source	2100 reflections with *I* > 2σ(*I*)
mirror	*R*_int_ = 0.026
Detector resolution: 15.9149 pixels mm^-1^	θ_max_ = 70.0°, θ_min_ = 4.4°
ω scans	*h* = −4→8
Absorption correction: multi-scan (*CrysAlis PRO*; Oxford Diffraction, 2009)	*k* = −12→12
*T*_min_ = 0.469, *T*_max_ = 0.524	*l* = −24→24
9086 measured reflections	

### Refinement

**Table:**

Refinement on *F*^2^	Secondary atom site location: difference Fourier map
Least-squares matrix: full	Hydrogen site location: inferred from neighbouring sites
*R*[*F*^2^ > 2σ(*F*^2^)] = 0.031	H-atom parameters constrained
*wR*(*F*^2^) = 0.109	*w* = 1/[σ^2^(*F*_o_^2^) + (0.0455*P*)^2^ + 0.2775*P*] where *P* = (*F*_o_^2^ + 2*F*_c_^2^)/3
*S* = 1.20	(Δ/σ)_max_ < 0.001
2373 reflections	Δρ_max_ = 0.15 e Å^−^^3^
172 parameters	Δρ_min_ = −0.26 e Å^−^^3^
0 restraints	Absolute structure: Flack (1983), 864 Friedel pairs
Primary atom site location: structure-invariant direct methods	Flack parameter: 0.01 (2)

### Special details

**Table:**

Experimental. Empirical absorption correction using spherical harmonics, implemented in SCALE3 ABSPACK scaling algorithm. CrysAlisPro (Oxford Diffraction, 2009)
Geometry. All esds (except the esd in the dihedral angle between two l.s. planes) are estimated using the full covariance matrix. The cell esds are taken into account individually in the estimation of esds in distances, angles and torsion angles; correlations between esds in cell parameters are only used when they are defined by crystal symmetry. An approximate (isotropic) treatment of cell esds is used for estimating esds involving l.s. planes.
Refinement. Refinement of *F*^2^ against ALL reflections. The weighted *R*-factor *wR* and goodness of fit *S* are based on *F*^2^, conventional *R*-factors *R* are based on *F*, with *F* set to zero for negative *F*^2^. The threshold expression of *F*^2^ > σ(*F*^2^) is used only for calculating *R*-factors(gt) *etc*. and is not relevant to the choice of reflections for refinement. *R*-factors based on *F*^2^ are statistically about twice as large as those based on *F*, and *R*- factors based on ALL data will be even larger.

### Fractional atomic coordinates and isotropic or equivalent isotropic displacement parameters (Å^2^)

**Table:**

	*x*	*y*	*z*	*U*_iso_*/*U*_eq_	
C1	0.4506 (5)	1.0038 (3)	0.44438 (13)	0.0628 (7)	
C2	0.2775 (5)	1.0218 (3)	0.48079 (15)	0.0783 (9)	
H2	0.2568	1.1009	0.5038	0.094*	
C3	0.1355 (6)	0.9220 (4)	0.48286 (16)	0.0815 (9)	
H3	0.0200	0.9334	0.5080	0.098*	
C4	0.1626 (5)	0.8067 (3)	0.44834 (16)	0.0785 (8)	
H4	0.0656	0.7401	0.4495	0.094*	
C5	0.3367 (5)	0.7894 (3)	0.41138 (14)	0.0673 (7)	
H5	0.3547	0.7109	0.3877	0.081*	
C6	0.4835 (4)	0.8867 (2)	0.40921 (12)	0.0559 (6)	
C7	0.6754 (4)	0.8661 (3)	0.37349 (13)	0.0602 (6)	
H7	0.7957	0.8931	0.3979	0.072*	
C8	0.6969 (4)	0.8680 (2)	0.30113 (13)	0.0571 (6)	
H8	0.8281	0.8969	0.2845	0.069*	
C9	0.5200 (4)	0.9026 (2)	0.25827 (12)	0.0519 (5)	
C10	0.2091 (4)	0.8095 (2)	0.20952 (11)	0.0485 (5)	
C11	0.0848 (4)	0.7002 (2)	0.21366 (14)	0.0591 (6)	
H11	0.1203	0.6288	0.2405	0.071*	
C12	−0.0942 (5)	0.6958 (3)	0.17779 (15)	0.0719 (8)	
H12	−0.1769	0.6213	0.1803	0.086*	
C13	−0.1475 (5)	0.8020 (3)	0.13874 (14)	0.0737 (8)	
H13	−0.2655	0.7993	0.1142	0.088*	
C14	−0.0262 (5)	0.9114 (3)	0.13617 (14)	0.0734 (8)	
H14	−0.0652	0.9840	0.1106	0.088*	
C15	0.1546 (5)	0.9176 (3)	0.17082 (13)	0.0618 (6)	
H15	0.2366	0.9924	0.1680	0.074*	
Cl1	0.63034 (15)	1.12873 (8)	0.44179 (4)	0.0905 (3)	
N1	0.3914 (3)	0.80345 (18)	0.24575 (10)	0.0524 (5)	
H1	0.4229	0.7267	0.2616	0.063*	
O1	0.6959 (3)	0.74497 (17)	0.33669 (10)	0.0666 (5)	
O2	0.5063 (3)	1.01692 (16)	0.23815 (11)	0.0732 (6)	

### Atomic displacement parameters (Å^2^)

**Table:**

	*U*^11^	*U*^22^	*U*^33^	*U*^12^	*U*^13^	*U*^23^
C1	0.0775 (18)	0.0568 (13)	0.0540 (12)	0.0057 (14)	−0.0038 (13)	0.0006 (12)
C2	0.100 (2)	0.0729 (19)	0.0619 (15)	0.0152 (19)	0.0041 (16)	−0.0041 (14)
C3	0.074 (2)	0.098 (2)	0.0730 (17)	0.004 (2)	0.0099 (16)	0.0101 (17)
C4	0.076 (2)	0.082 (2)	0.0773 (18)	−0.0094 (18)	−0.0062 (17)	0.0141 (17)
C5	0.0738 (19)	0.0615 (15)	0.0667 (15)	−0.0019 (15)	−0.0138 (15)	0.0035 (13)
C6	0.0609 (15)	0.0507 (12)	0.0561 (12)	0.0041 (13)	−0.0142 (11)	0.0003 (11)
C7	0.0592 (16)	0.0489 (12)	0.0725 (14)	0.0062 (13)	−0.0135 (12)	−0.0100 (12)
C8	0.0577 (15)	0.0389 (11)	0.0748 (15)	0.0054 (12)	−0.0031 (12)	−0.0085 (11)
C9	0.0551 (14)	0.0364 (10)	0.0640 (13)	−0.0004 (11)	−0.0007 (11)	−0.0090 (10)
C10	0.0525 (14)	0.0422 (11)	0.0508 (11)	−0.0012 (11)	0.0024 (10)	−0.0061 (10)
C11	0.0620 (16)	0.0448 (12)	0.0704 (14)	−0.0027 (12)	0.0007 (12)	−0.0039 (11)
C12	0.0711 (19)	0.0627 (16)	0.0817 (18)	−0.0081 (15)	0.0004 (15)	−0.0113 (14)
C13	0.0618 (17)	0.089 (2)	0.0700 (16)	−0.0041 (18)	−0.0101 (14)	−0.0109 (15)
C14	0.0758 (19)	0.0778 (18)	0.0666 (15)	0.0019 (17)	−0.0127 (14)	0.0110 (14)
C15	0.0706 (17)	0.0517 (13)	0.0630 (13)	−0.0048 (13)	−0.0055 (13)	0.0068 (12)
Cl1	0.1070 (7)	0.0629 (4)	0.1016 (5)	−0.0142 (5)	0.0117 (5)	−0.0220 (4)
N1	0.0576 (12)	0.0362 (8)	0.0632 (11)	0.0002 (9)	−0.0047 (9)	0.0007 (8)
O1	0.0723 (12)	0.0446 (9)	0.0830 (12)	0.0140 (9)	−0.0153 (10)	−0.0070 (9)
O2	0.0799 (13)	0.0359 (8)	0.1039 (14)	−0.0044 (9)	−0.0200 (11)	0.0020 (9)

### Geometric parameters (Å, °)

**Table:**

C1—C2	1.381 (4)	C8—H8	0.9800
C1—C6	1.391 (3)	C9—O2	1.221 (3)
C1—Cl1	1.734 (3)	C9—N1	1.337 (3)
C2—C3	1.378 (5)	C10—C11	1.377 (3)
C2—H2	0.9300	C10—C15	1.387 (4)
C3—C4	1.364 (5)	C10—N1	1.420 (3)
C3—H3	0.9300	C11—C12	1.397 (4)
C4—C5	1.391 (5)	C11—H11	0.9300
C4—H4	0.9300	C12—C13	1.374 (4)
C5—C6	1.383 (4)	C12—H12	0.9300
C5—H5	0.9300	C13—C14	1.364 (4)
C6—C7	1.483 (4)	C13—H13	0.9300
C7—O1	1.432 (3)	C14—C15	1.395 (4)
C7—C8	1.472 (4)	C14—H14	0.9300
C7—H7	0.9800	C15—H15	0.9300
C8—O1	1.429 (3)	N1—H1	0.8600
C8—C9	1.504 (4)		
			
C2—C1—C6	121.0 (3)	C7—C8—H8	115.6
C2—C1—Cl1	119.9 (2)	C9—C8—H8	115.6
C6—C1—Cl1	119.1 (2)	O2—C9—N1	126.0 (2)
C3—C2—C1	119.6 (3)	O2—C9—C8	117.9 (2)
C3—C2—H2	120.2	N1—C9—C8	116.1 (2)
C1—C2—H2	120.2	C11—C10—C15	120.0 (2)
C4—C3—C2	120.7 (3)	C11—C10—N1	116.7 (2)
C4—C3—H3	119.7	C15—C10—N1	123.3 (2)
C2—C3—H3	119.7	C10—C11—C12	120.5 (3)
C3—C4—C5	119.5 (3)	C10—C11—H11	119.8
C3—C4—H4	120.3	C12—C11—H11	119.8
C5—C4—H4	120.3	C13—C12—C11	119.7 (3)
C6—C5—C4	121.2 (3)	C13—C12—H12	120.1
C6—C5—H5	119.4	C11—C12—H12	120.1
C4—C5—H5	119.4	C14—C13—C12	119.6 (3)
C5—C6—C1	118.0 (3)	C14—C13—H13	120.2
C5—C6—C7	121.7 (2)	C12—C13—H13	120.2
C1—C6—C7	120.2 (2)	C13—C14—C15	121.9 (3)
O1—C7—C8	58.96 (16)	C13—C14—H14	119.1
O1—C7—C6	117.0 (2)	C15—C14—H14	119.1
C8—C7—C6	124.5 (2)	C10—C15—C14	118.4 (3)
O1—C7—H7	114.8	C10—C15—H15	120.8
C8—C7—H7	114.8	C14—C15—H15	120.8
C6—C7—H7	114.8	C9—N1—C10	127.89 (19)
O1—C8—C7	59.12 (16)	C9—N1—H1	116.1
O1—C8—C9	119.1 (2)	C10—N1—H1	116.1
C7—C8—C9	120.1 (2)	C8—O1—C7	61.92 (15)
O1—C8—H8	115.6		

### Hydrogen-bond geometry (Å, °)

**Table:**

*D*—H···*A*	*D*—H	H···*A*	*D*···*A*	*D*—H···*A*
N1—H1···O1	0.86	2.38	2.801 (3)	111
N1—H1···O2^i^	0.86	2.16	2.973 (2)	158

Symmetry codes: (i) −*x*+1, *y*−1/2, −*z*+1/2.
